# Developing a national pharmaceutical research strategy in Lebanon: opportunities to bridge the gaps and reach the goals

**DOI:** 10.1186/s40545-022-00485-1

**Published:** 2022-11-17

**Authors:** Marwan Akel, Aline Hajj, Hala Sacre, Rony M. Zeenny, Chadia Haddad, Pascale Salameh

**Affiliations:** 1 INSPECT-LB (Institut National de Santé Publique, d’Épidémiologie Clinique et de Toxicologie-Liban), Beirut, Lebanon; 2 School of Pharmacy, Lebanese International University, Beirut, Lebanon; 3 Faculté de Pharmacie, Université Laval, Québec, Canada; 4 Oncology Division, CHU de Québec Université Laval Research Center, Québec, Canada; 5 Laboratoire de Pharmacologie, Pharmacie Clinique et Contrôle de Qualité des Médicament, Faculty of Pharmacy, Université Saint-Joseph de Beyrouth, Beirut, Lebanon; 6 Department of Pharmacy, American University of Beirut Medical Center, Beirut, Lebanon; 7 School of Medicine, Lebanese American University, Byblos, Lebanon; 8 Research Department, Psychiatric Hospital of the Cross, P.O. Box 60096, Jal Eddib, Lebanon; 9 School of Health Sciences, Modern University for Business and Science, Beirut, Lebanon; 10 Faculty of Pharmacy, Lebanese University, Hadath, Lebanon; 11 Department of Primary Care and Population Health, University of Nicosia Medical School, 2417 Nicosia, Cyprus

**Keywords:** Strategy, Pharmaceutical research, Research policy, Projects, Research

## Abstract

Pharmaceutical research can be structured into clear national strategies that optimize patient health and foster innovation. The objectives of this document are to assess the need for a national pharmaceutical research strategy based on the current situation in Lebanon, to identify the strengths and weaknesses of pharmaceutical research in Lebanon, and to suggest a pharmaceutical research strategy for Lebanon, including goals and objectives. In Lebanon, in the absence of a national health research policy, pharmaceutical research is conducted in academia or hospitals, although projects are the result of personal or team initiatives that should be organized to better serve the needs of the country. Many strengths of pharmaceutical research were identified, such as the pharmaceutical workforce and academics who are willing to contribute to research, while the implementation of the national pharmaceutical strategy represents an important opportunity to promote research. Among the weaknesses is the lack of research culture in some institutions and interinstitutional/interprofessional collaborations. Thus, the suggested strategy aims to structure pharmaceutical research in Lebanon, including the priorities towards which research is directed, the process by which research is conducted, and the workforce conducting research. It will mainly rely on the World Health Organization's interrelated goals (organization, priorities, capacity, standards, and translation). The implementation of the suggested pharmaceutical research strategy will only be achieved through the leadership of the pharmaceutical authorities and the collaboration of stakeholders.

## Background

### Health research strategies worldwide

Research strategies that organize and support research efforts are widely recommended to assess specific health needs, promote innovation, and make evidence-based decisions. In 2012, the World Health Organization (WHO) published its global research health strategy based on four pillars, i.e., the capacity optimization of health systems, priorities assessment to meet health needs, standards for good research practice, and translation of evidence into practice [1]. Furthermore, many national research strategies have been developed worldwide through initiatives of governmental or non-governmental bodies. For example, in Australia, the Association of Australian Medical Research Institutes recommended a National Health and Medical Research Strategy in November 2021, based on three main pillars: research (identifying research needs, aligning strategic investment, integrating research, and healthcare delivery), workforce (developing sustainable and rewarding careers), and funding (building coordinated and sustainable funding mechanisms) [2, 3]. In Canada, the Association of Faculties of Medicine of Canada (AFMC) suggested a National Health Research Strategy in a position paper inspired by the success and importance of research during the COVID-19 pandemic. According to the AFMC, the environment and infrastructure currently in place should be improved to secure collaboration across all sectors and allow Canadians to address national health needs and contribute to global solutions. It also stated that “the close integration of academic research with industry is critical in responding to pandemics but is also critical in many other areas of healthcare” [4]. Moreover, research strategies have also been suggested in some developing countries, such as South Africa [5] and Bangladesh [6].

### Health research in Lebanon

In Lebanon, research is conducted through academia or hospitals, with a solid research capacity pillared on its knowledgeable human capital. According to the Lebanese structure of higher education and its governing regulations (namely, law number 285/2014), universities should develop their research capabilities and have an organizational infrastructure for conducting research; in addition, the quality evaluations of academics and institutions are widely based on research productivity [7]. A detailed analysis of biomedical research showed that biomedical research outputs quickly recovered in Lebanon following a long war (1974–1992), mainly supported by uninterrupted activities in private higher education institutes [8]. Between 1985 and 2004, 1964 medical articles were counted over a two-decade period; however, the growth rate of publication drew a decline passing from 202% (1990–1994) to 55.3% (2000–2004) [9]: the four most productive specialties (anesthesiology, internal medicine, gynecology, and pediatrics) published 611 articles (31.1%), mainly in Beirut [9].

### Pharmaceutical research scope and strategies

Pharmaceutical research encompasses steps that start from the development of a pharmaceutical product, its trial in animals and humans, and its use among patients; it includes various disciplines, such as basic pharmacology and toxicology, medicinal chemistry, clinical pharmacology, toxicology, pharmacokinetics, pharmaceutics, pharmacoepidemiology, pharmacoeconomics, and pharmacogenetics. It also covers medication-related public health issues (antibiotic resistance, medication safety, and clinical toxicology), pharmacy education (initial, experiential, theoretical, and continuing), pharmacy workforce, and pharmacy governance. Pharmaceutical research further tackles the advancement of pharmacy practice; it strengthens pharmacist-provided services, builds the evidence base for developing and commissioning new services, improves patient care, and contributes to health service knowledge [10, 11]. Research related to pharmacy education, workforce, and governance is also part of pharmaceutical research. It facilitates the implementation, monitoring, and evaluation of drug policies and their impact on national health services; it contributes to the assessment of drug access, quality, and rational use (prescribing, dispensing, and socioeconomic aspects) [12].

Pharmaceutical research should be structured into clear national strategies that optimize patient health and foster innovation, prioritizing actions and evidence-based policy making, such as New Zealand [13]; another example includes the Pharmaceutical Strategy for Europe that includes innovation and development of new medications as a major pillar [14]. Moreover, strategies’ methods for drug research and development were detailed [15] in the literature [16]; they are also frequently used by pharmaceutical industry during the development of their innovative products [17].

### Pharmaceutical research Lebanese context

In Lebanon, in the absence of a national health research policy, all available projects result from personal initiatives. Research topics in health, including the pharmaceutical topics, are sometimes duplicated, unrelated, or non-continuous. In addition, pharmaceutical companies contribute to pharmaceutical research in the country according to their agendas and priorities. Thus, even if some universities and hospitals have research policies and departments, a national research policy is essential to help decision-makers regulate and promote research in Lebanon, establish mandatory and nationally helpful research within institutions, and improve patient health and quality of life.

The Order of Pharmacists of Lebanon (OPL), the national pharmaceutical authority in Lebanon, has already been leading on strategic matters related to the pharmaceutical sector. In May 2022, it developed the National Pharmaceutical Strategy (NPS) in collaboration with all the relevant stakeholders [18]; the NPS will make an important part of the National Health Strategy (NHS) that is being developed by the Lebanese Ministry of Public Health. Its implementation plan is now being approved by the authorities. The NPS strategy includes many items that show the need to organize and support pharmaceutical research and report the absence of a national research strategy for pharmacy and pharmaceutical products.

### Objectives

This document aims:

- To assess the need for a National Pharmaceutical Research Strategy (NPRS) based on the current situation in Lebanon (bibliometric analysis and available research);
- To identify the strengths and weaknesses of pharmaceutical research in Lebanon;
- To suggest a pharmaceutical research strategy for Lebanon, including goals and objectives.

## Situation assessment of pharmaceutical research in Lebanon

### Pharmaceutical research productivity in Lebanon

A specific search on the pharmaceutical research conducted on Scopus on August 9, 2022, using the keywords (AFFILCOUNTRY (Lebanon) AND ABS ((pharma*OR medication OR medicine OR drug OR treatment OR therapy))) returned 9299 documents. Publications in the pharmaceutical field started early in 1934, but the numbers began to increase significantly after 1990 (the end of the civil war). These numbers continued to grow exponentially, with the last 5 years showing substantial figures, i.e., 616 in 2017 up to 1027 in 2018. Among the top ten contributing authors, two are pharmacists (ranking second and fifth), while eight are physicians. Documents were classified according to the following areas: Medicine (*n* = 6552; 46.9%), Biochemistry, Genetics and Molecular Biology (*n* = 1679; 12%), Pharmacology, Toxicology and Pharmaceutics (*n* = 943; 6.7%), and Immunology and Microbiology (*n* = 520; 3.7%).

### Research conducted by pharmacists in Lebanon

In Lebanon, although research by pharmacists is valuable and promoted [19], research is conducted by academic and hospital pharmacists mainly, with low participation rates of pharmacists in the community, where several barriers were identified [20, 21], including lack of time, financial resources, support, knowledge, and education. A review of the literature has concluded that 792 studies [731 (92.3%) observational studies, 29 (3.7%) interventional studies, and 61 (7.7%) clinical trials] have been conducted by at least one academic pharmacist in Lebanon between 2010 and 2018. The most addressed areas/specialties were infectious diseases (10%), oncology (8.2%), public health (7.8%), pharmacy practice (7.3%), genetics (6.8%), behavioral psychology (6.7%), cardiovascular diseases (5.8%), and psychiatry (5.2%) [20].

### Pharmaceutical research in Lebanese institutions

In university hospitals, pharmacists conduct research in collaboration with physicians, especially when clinical research involves medications. A bibliometric study showed high productivity in the three largest university hospitals in Beirut, i.e., the American University of Beirut Medical Center, Hôtel-Dieu de France Hospital, and Saint Georges University Medical Center [9]. As for the academic sector, there are five universities teaching pharmacy in Lebanon; research in these institutions is not a requirement, except for tenure track instructors, where research productivity is considered for promotion. However, the recent introduction of accreditation to all universities made research paramount, particularly for graduate studies programs [20].

In addition, to make evidence-based decision and work on clear strategic plans [22], the OPL has also been involved in several research projects, mainly in collaboration with the Institut National de Santé Publique, Epidémiologie Clinique et Toxicologie—Liban (INSPECT-LB) and other research teams. These projects were related to pharmacy workforce and practice, assessing workforce education [23], graduates planning [24, 25], continuing education [26] and practice [27–32]. Some of these projects could be used for decision-making that improves pharmacy practice [33–35]. Many research projects were also conducted during the COVID-19 pandemic and were helpful in assessing pharmacists’ readiness and the need for optimization [36–40].

## SWOC analysis of pharmaceutical research in Lebanon

There is research in different pharmacy sectors in Lebanon, but a lack of research projects mapping and the absence of a database of national projects makes it difficult to optimize the available research according to the country needs. The Strengths, Weaknesses, Opportunities, and Challenges (or Threats) (SWOC) were analyzed and summarized, based on the conducted situation assessment (Table 1).

**Table 1:** SWOC analysis of pharmaceutical research in Lebanon

Strengths - The pharmaceutical workforce is willing to contribute to research - Some researchers are working on pharmaceutical topics and becoming increasingly active - Researchers have access to internal funds from their institutions and sometimes international funds - Many academic institutions offer research-related master’s degrees with good research practice and methods taught - Many assessment projects related to pharmaceutical research are being conducted - Can we discuss that researchers in Lebanon have good collaborations with international research teams? -Good visibility of researchers in international conferences/meetings?	Opportunities - The implementation of the National Pharmaceutical Strategy is pending - Many academic and hospital institutions require research in their accreditation standards - Funding by international agencies is available for Lebanese researchers but might need to be consolidated - The pharmaceutical research culture is being promoted in many institutions, but it needs consolidation, especially to expose potential young researchers (students, research assistants, etc.) - Interprofessional Education and Collaboration (IPE/IPC) research should be implemented - There is a need for more collaboration within the same majors/domains (between research teams or laboratories of the same institution, for example)
Weaknesses - Researchers’ workforce in Lebanon is not mapped - Research culture is not established in all institutions; many researchers are not valued - There is no official competency framework for researchers in Lebanon - Research in Lebanon is fragmented and not included in a strategy - The conducted research projects do not always answer priority needs in Lebanon - The impact of the research conducted in Lebanon on the decisions related to public and clinical health is not clear - There is a general lack of funding for research	Challenges - Interinstitutional/interprofessional collaborations are hard to apply (competitiveness, people not used to working together, etc.) - Conducting large research projects with substantial funds is sometimes hard to manage - Political influences and the socioeconomic crisis might affect researchers’ decisions to leave Lebanon - Establishing priorities should be objective and based on the available information (evidence-based) that is sometimes not readily available - There is no clear research axes/strategies at the institution/national level (even if some exceptions are noted for some institutions)

## Suggested strategy

Based on the situation assessment and the SWOC analysis, the new research strategy was suggested. Its purpose, vision, core values, suggested goals based on international guidelines (mainly the WHO), and suggested objectives for every goal were developed.

### Purpose, vision, and core values for the strategy

This national pharmaceutical research strategy aims to align the pharmaceutical research in Lebanon, including the priorities towards which research is directed, the process by which research is conducted, and the workforce conducting research. Its vision is to reach a point where Lebanon would be recognized as a regional hub for pharmaceutical research, contributing to patient quality of care and health. As for the core values, teamwork, integrity, quality, equity, impartiality, and sustainability are suggested.

### Suggested pharmaceutical research strategy goals

The suggested national pharmaceutical research strategy would mainly rely on the WHO's five inter-related goals [1] and would be based on the available evidence and SWOC analysis. Five main goals were defined: culture, prioritization, workforce, good practice and policy making (Table 2). Identifying the missing links and bridging the gaps between the current situation and the future vision is key to achieving these goals through smart objectives (Table 3).

**Table 2:** Suggested pharmaceutical research strategy goals based on WHO principles

WHO research strategy goals	National pharmaceutical research strategy goals
Strengthening of the research culture in WHO so that the Organizations can lead by example	Establishing a national research culture in the pharmaceutical sector
Focusing research globally on priority health needs	Focusing pharmaceutical research on national priority health needs
Helping strengthen national systems for health research	Helping institutions and workforce in developing sustainable pharmaceutical research
Promoting good practice in research strategy, setting norms and standards	Promoting good practice in research, setting norms and standards at the national level
Strengthening links between health research and health policy and practice	Strengthening links between pharmaceutical research and health policy and practice

**Table 3:** Suggested objectives per goal

Goal 1: Establishing a National Research Culture in the Pharmaceutical sector It is suggested that pharmaceutical authorities take the following actions to keep pace with a changing research environment and communicate better research activities at the national level: • Establishing a national center for pharmaceutical research to assess, monitor, and conduct pharmaceutical research in the country • Raising resources to support strategy development and implementation • Keeping abreast of health-related developments and information in Lebanon through a national pharmaceutical research observatory • Networking and regularly exchanging with the national and international research community • Improving scientific communication and dissemination of research results • Developing an open-access repository of databases and results of all research
Goal 2: Focusing Pharmaceutical Research on National Priority Health Needs The goal is to champion research on priority health needs. The strategy would help identify research priorities and mobilize the response. The range of health challenges is broad, and agreeing on priorities is not always easy. Actions to be taken by pharmaceutical authorities to achieve the priority goal include: • Synthesizing data on gaps in national and global pharmaceutical research • Convening consultations to identify the priorities and funding for research based on available information, gaps in data, and health priorities • Reporting on national priorities and resources for pharmaceutical research • Developing research agendas for priority areas • Advocating support for research on national priorities • Improving the coherence of research activities by reviewing research agendas, including criteria for initiating, adjusting, and ending research programs
Goal 3: Helping Institutions and Workforce in developing a Sustainable Pharmaceutical Research Actions to be taken by pharmaceutical authorities, in collaboration with other stakeholders, to achieve this goal include: •Mapping the research workforce in Lebanese institutions, assessing their plans, and projecting their future directions •Attracting and retaining young and brilliant researchers and fostering potential future leaders in pharmaceutical research •Promoting capacity building related to research in academia and hospitals and improving research competencies •Supporting research projects that fall within the scope of national priorities •Promoting interdisciplinary, inter-university, interinstitutional, and interprofessional research projects •Reviewing the arrangements for working with partners and seeking partners from all sectors that impact sustainable pharmaceutical research
Goal 4: Promoting Good Practice in Research and Setting Norms/Standards at the National Level Actions to be taken by pharmaceutical authorities, in collaboration with other stakeholders (particularly the Ministry of Public Health in Lebanon), to achieve this goal include: • Developing a code of good research practice to be added to pharmacy curricula • Strengthening ethical standards and peer review, using evidence to develop guidelines, and reviewing existing policies in the light of new evidence • Promoting ethics committees’ practice in institutions under the authority of competent ministries • Developing and implementing quality standards for the accreditation of research laboratories
Goal 5: Strengthening Links Between Pharmaceutical Research and Health Policy and Practice Actions to be taken by pharmaceutical authorities, in collaboration with other stakeholders (particularly MOPH), to achieve this goal include: • Establishing a national committee that suggests relevant pharmaceutical policies based on research results • Ensuring research results are transformed into health policies, as necessary, in collaboration with the involved authorities • Promoting the culture of evidence-based decisions • Assess the impact of pharmaceutical research on patient health in Lebanon, using appropriate methods (e.g., registries, electronic platforms)

The principal actor would be the national pharmaceutical authority (OPL), which should work on implementing the strategy in collaboration with the involved stakeholders (academia, hospitals, the CNRS-L, the National Institute of Public Health, Clinical Epidemiology, and Toxicology—Lebanon (INSPECT-LB), the ministries of public health and higher education, the WHO national office, non-governmental organizations, and pharmaceutical companies). Priorities, timelines, responsible parties, and indicators would then be defined, and the implementation would start.

## Discussion

This document is the first pharmaceutical research strategy suggested in Lebanon, in line with the WHO recommendations and goals. To be put in place, this strategy requires a consensus between the various stakeholders, added to a detailed plan that displays activities, responsible parties, involved stakeholders, priority level, timelines, and performance indicators (input, output, and impact tools would help evaluate the achievement of fixed goals in a timely manner).

The national pharmaceutical association, OPL, should consider the project a priority. Research culture and ethics would be spread through appropriate activities involving practicing pharmacists and pharmacy students in collaboration with academia. From the assessment aspect, there is a need to evaluate pharmaceutical topics rarely addressed by Lebanese researchers. Research workforce and teams that should be mapped to estimate the needs in fields of expertise that require bridging gaps in research topics.

The suggested strategy compares well with those of other countries, with adaptation to the Lebanese context, taking the positive facets and values from that of the UK [41], Canada [42], and Australia [2], with less emphasis on local sources of funding. Expectedly, it is closer to that of India, a developing country with some difficulties similar to those of Lebanon [43]. Lessons learned from international strategies would help the Lebanese authorities anticipate the barriers and adjust accordingly.

Many roadblocks are expected, such as difficulties in applying the core values (teamwork, integrity, quality, equity, impartiality, and sustainability). These difficulties extend to all health aspects, where partnership culture and interprofessional collaboration remain to be established. There is also a need for expertise to manage large and top-notch projects, which is possible through international partnerships. Moreover, the political influence and socioeconomic hardship are also expected to slow the implementation process, mainly because of a brain drain and the lack of motivation of local researchers in a country that has been enduring due to geopolitical (wars, refugees, and hegemony) and internal (corruption and lack of expertise, consensus, and resources) predicaments.

Nevertheless, many facilitators and opportunities are foreseen, such as the application of national strategies in related fields (mainly the national health strategy by the MOPH and the national pharmaceutical strategy by the OPL [18]), the international funding to which Lebanese researchers can apply in association with international collaborators, and the accreditation culture that indirectly affects research quality in educational institutions and hospitals. Local academia (mainly the five universities with a pharmacy program), university hospitals (some of which harbor pharmacy residency programs), and research-oriented institutions/associations, such as the CNRS-L and INSPECT-LB, are potential collaborators in the implementation of the strategy. A helping hand would also be expected from the local or multinational pharmaceutical industry, which contributes to the local development of research through their outreach and promotion of science [44, 45]; in this case, the conflict of interest should be closely watched. International instances, such as the WHO and the FIP, could be solicited, mainly for capacity-building.

## Conclusion

Although pharmaceutical research is carried out in Lebanon, several gaps exist. Hence, implementing a national pharmaceutical research strategy is crucial and would only be achieved through the leadership of relevant pharmaceutical authorities and the collaboration of stakeholders. Bridging the gaps and achieving the stated vision is expected to optimize patient health through the rational use of medications in disease prevention and treatment and ameliorate the status of the pharmaceutical workforce in a country plagued by daunting problems.

## Data Availability

Not applicable.

## Abbreviations

CNRS-L: Lebanese National Council for Scientific Research
FIP: International Pharmacy Federation
IRB: Institutional Review Board
MOPH: Ministry of Public Health
OPL: Order of Pharmacists of Lebanon
REC: Research and Ethics Committee
WHO: World Health Organization

## References

[1] WHO. The WHO strategy on research for health. 2012:62. https://www.who.int/publications/i/item/9789241503259. Accessed 26 Sept 2022.

[2] Australian Government. Australian medical research and innovation strategy 2021–2026 and the related Australian medical research and innovation priorities consultation report. 44. https://www.health.gov.au/sites/default/files/documents/2021/11/australian-medical-research-and-innovation-strategy-2021-2026-and-the-related-priorities-consultation-report_1.pdf. Accessed 26 Sept 2022

[3] Association of Australian. Medical Research Institutes (AAMRI). White paper: a national health and medical research strategy—AAMRI—Reports and Other Sector Info. https://www.aamri.org.au/wp-content/uploads/2021/11/Australias-Missing-Link-A-National-Health-and-Medical-Research-Strategy.pdf. Accessed 26 Sept 2022.

[4] The Association of Faculties of Medicine of Canada (AFMC). A national integrated health research strategy—position paper. The Association of Faculties of Medicine of Canada (AFMC); 2021:5. https://www.afmc.ca/sites/default/files/pdf/AFMC_Research_Position_Paper_EN.pdf. Accessed 26 Sept 2022.

[5] Republic of South Africa-Department of Health. National health research strategy: research priorities for South Africa 2021–2024 (Revised). 2021:22. https://www.health.gov.za/wp-content/uploads/2022/05/National-Health-Research-Priorities-2021-2024.pdf. Accessed 26 Sept 2022.

[6] Directorate General of Health Services. National health strategy. Ministry of Health and Family Welfare; 33. https://www.bmrcbd.org/Bulletin/NOC/National%20Health%20Research%20Stratgey.pdf. Accessed 26 Sept 2022

[7] Education AJd. Culture Executive Agency (EACEA). Teaching reading in Europe: contexts, policies, and practices. Education, audiovisual and culture executive agency P9 Eurydice Avenue du Bourget 1 (BOU2) B-1140 Brussels. 2011;10:60196.

[8] Nisrine BT, Ghazi OT (2009). Bibliometric analyses of biomedical research outputs in Lebanon and the United Arab Emirates [1988–2007]. Saudi Med J.

[9] Mazboudi M (2010). Medical research productivity of Lebanon: a bibliometric study of papers indexed in Medline, 1985–2004. J Tunis Med.

[10] Obaid D, El-Dahiyat F, Babar ZUD (2022). Pharmacy practice and clinical pharmacy research in the Middle East: a scoping review of studies from Bahrain, Iraq, Jordan, Kuwait, Lebanon, Oman, Palestine, Qatar, Saudi Arabia, Syria, United Arab Emirates, and Yemen. J Pharm Policy Pract.

[11] Awaisu A, Alsalimy N (2015). Pharmacists' involvement in and attitudes toward pharmacy practice research: a systematic review of the literature. J Res Soc Admin Pharm.

[12] WHO. How to develop and implement a National drug policy—2nd Ed.; 2001:96. https://www.who.int/publications-detail-redirect/924154547X. Accessed 26 Sept 2022.

[13] Francis S (2014). Identifying priority medicines policy issues for New Zealand: a general inductive study. J BMJ Open.

[14] Garattini S, Natsis Y, Banzi R (2021). Pharmaceutical strategy for Europe: reflections on public health-driven drug development, regulation, and policies. J Front Pharmacol..

[15] Sedlacek H-H, Sapienza AM, Eid V (2008). Ways to successful strategies in drug research and development.

[16] Kuhlmann J (1999). Alternative strategies in drug development: clinical pharmacological aspects. Int J Clin Pharmacol Ther.

[17] Margarido RJF (2018). Data-driven process improvement in the pharmaceutical industry.

[18] Sacre H, Hamra R, Hassoun C, Karam C, Rifai O, P S. Towards a national pharmaceutical strategy in Lebanon: a commitment and a call to action to ensure access to quality and safe medications in Lebanon for all; 2022. 10.13140/RG.2.2.14429.49120.

[19] Hallit S, Hajj A, Sacre H (2019). Emphasizing the role of pharmacist as a researcher: the Lebanese order of pharmacists’ perspective. J Res Pharm Pract.

[20] Hajj A, Sacre H, Salameh P. Pharmacy education, practice, and research in Lebanon. In: Handbook of medical and health sciences in developing countries: education, practice, and research (Editor: Yaser Al-Worafi). Springer. 2021. https://www.researchgate.net/publication/353980394_Handbook_of_Medical_and_Health_Sciences_in_Developing_Countries. Accessed 26 Sept 2022

[21] Zeidan RK, Hallit S, Zeenny RM, Salameh P (2019). Lebanese community-based pharmacists’ interest, practice, knowledge, and barriers towards pharmacy practice research: a cross-sectional study. J Saudi Pharmaceutical Journal.

[22] Hallit S, Sacre H, Salameh P (2019). Role of a professional organization in promoting and conducting research: the Lebanese Order of Pharmacists’ experience. Int J Pharm Pract.

[23] Zeenny RM, Akel M, Hajj A, Sacre H, Hallit S, Salameh P (2021). Descriptive assessment of graduates' perceptions of pharmacy-related competencies based on the Lebanese pharmacy core competencies framework. J Pharm Pract..

[24] Sacre H, Hallit S, Hajj A, Zeenny RM, Sili G, Salameh P (2019). The pharmacy profession in a developing country: challenges and suggested governance solutions in Lebanon. J Res Pharm Pract.

[25] Hallit S, Sacre H, Hajj A, Sili G, Zeenny RM, Salameh P (2019). Projecting the future size of the Lebanese pharmacy workforce: forecasts until the year 2050. Int J Pharm Pract.

[26] Sacre H, Tawil S, Hallit S, Hajj A, Sili G, Salameh P (2019). Attitudes of Lebanese pharmacists towards online and live continuing education sessions. J Pharm Pract..

[27] Hallit S, Sacre H, Zeenny R, Hajj A, Sili G, Salameh P (2019). Credentialing and recognition of pharmacy specializations: the Lebanese order of pharmacists initiative. ACCP Int Clin Pharm.

[28] Fahs IM, Hallit S, Rahal MK, Malaeb D (2018). The community pharmacist’s role in reducing cardiovascular risk factors in lebanon: a longitudinal study. Med Princ Pract.

[29] Mouhtadi BB, Alame MM, Malaeb B, Hallit S, Salameh P, Malaeb D (2018). Physician-community pharmacist collaborative care in diabetes management: a pilot study. J Drug Assess.

[30] Hallit S, Hajj A, Shuhaiber P (2019). Order of Pharmacists of Lebanon scientific committee—medication safety subcommittee. Medication safety knowledge, attitude, and practice among hospital pharmacists in Lebanon. J Eval Clin Pract..

[31] Domiati S, Sacre H, Lahoud N, Sili G, Salameh P (2018). Knowledge of and readiness for medication therapy management among community pharmacists in Lebanon. Int J Clin Pharm.

[32] Hallit S, Zeidan RK, Saade S (2020). Knowledge, attitude and practice of lebanese community pharmacists toward chronic obstructive pulmonary disease. J Epidemiol Glob Health.

[33] Badro DA, Sacre H, Hallit S, Amhaz A, Salameh P (2020). Good pharmacy practice assessment among community pharmacies in Lebanon. J Pharm Pract..

[34] Rahme D, Lahoud N, Sacre H, Akel M, Hallit S, Salameh P (2020). Work fatigue among Lebanese community pharmacists: prevalence and correlates. J Pharm Pract..

[35] Hajj A, Sacre H, Hallit S, Zeenny RM, Sili G, Salameh P (2020). Prescription and dispensing guidelines in Lebanon: initiative of the Order of Pharmacists of Lebanon. J Pharm Policy Pract.

[36] Zeenny RM, Ramia E, Akiki Y, Hallit S, Salameh P (2020). Assessing knowledge, attitude, practice, and preparedness of hospital pharmacists in Lebanon towards COVID-19 pandemic: a cross-sectional study. J Pharm Policy Pract.

[37] Zeenny RM, Dimassi A, Sacre H (2021). A cross-sectional survey on community pharmacists readiness to fight COVID-19 in a developing country: knowledge, attitude, and practice in Lebanon. J Pharm Policy Pract.

[38] Sakr F, Akiki Z, Dabbous M, Salameh P, Akel M (2021). The role of pharmacists in providing immunization to the general population: are Lebanese pharmacists ready for this role?. Pharm Pract.

[39] Safwan J, Cherfan M, Dabbous M (2022). Faculty perceptions on online education in a school of pharmacy during the COVID-19 pandemic. Pharm Educ.

[40] Bou-Saba AW, Kassak KM, Salameh P (2022). Public views of community pharmacy services during the COVID-19 pandemic: a national survey. J Pharm Policy Pract.

[41] Walley T, Earl-Slater A, Haycox A, Bagust A (2000). An integrated national pharmaceutical policy for the United Kingdom?. BMJ.

[42] Morgan SG, Gagnon M-A, Mintzes B, Lexchin J (2016). A better prescription: advice for a national strategy on pharmaceutical policy in Canada. Healthc Policy.

[43] Sindkhedkar M, Jagtap S, Shah C, Palle VP (2020). Pharmaceutical research in India: current status and opportunities. Proc Indian Natl Sci Acad.

[44] McHugh M, Hayes S, Tajber L, Ryan L (2022). Medicine maker: an outreach activity for pharmaceutical manufacturing and health literacy. J Chem Educ.

[45] Yeung AWK, Atanasov AG, Sheridan H (2021). Open innovation in medical and pharmaceutical research: a literature landscape analysis. Front Pharmacol.

